# Gender Moderates the Partial Mediation of Impulsivity in the Relationship Between Psychiatric Distress and Problematic Online Gaming: Online Survey

**DOI:** 10.2196/10784

**Published:** 2019-03-19

**Authors:** Wenliang Su, Orsolya Király, Zsolt Demetrovics, Marc N Potenza

**Affiliations:** 1 Department of Applied Psychology School of Humanities and Social Sciences Fuzhou University Fuzhou China; 2 Institute of Psychology Eötvös Loránd University Budapest Hungary; 3 Department of Psychiatry School of Medicine Yale University New Haven, CT United States; 4 Child Study Center School of Medicine Yale University New Haven, CT United States; 5 Department of Neuroscience School of Medicine Yale University New Haven, CT United States; 6 Connecticut Council on Problem Gambling School of Medicine Yale University New Haven, CT United States; 7 Connecticut Mental Health Center New Haven, CT United States

**Keywords:** internet, video games, addictive behavior, psychopathology, impulsivity, gender

## Abstract

**Background:**

Research has shown that some individuals can develop problematic patterns of online gaming, leading to significant psychological and interpersonal problems. Psychiatric distress and impulsivity have been suggested to contribute to problematic online gaming (POG).

**Objective:**

This study aimed to investigate the potential mediating or moderating mechanisms of impulsivity and gender-related differences in possible associations between psychiatric distress and POG.

**Methods:**

A total of 596 matched female and male participants, ranging in age from 14 to 38 years (mean 21.4, SD 4.5), were chosen from a large cross-sectional, nationwide Hungarian online gaming sample. Participants completed online questionnaires about self-reported impulsivity, psychiatric distress, and POG.

**Results:**

Psychiatric distress directly predicted POG, and impulsivity partially mediated the relationship between psychiatric distress and POG. However, this mediation effect was found only for the impatience factor of impulsivity. Impulsivity did not moderate the relationship between psychiatric distress and POG. A moderating effect of gender was not found in the direct relationship between psychiatric distress and POG. However, a moderated mediation analysis revealed that impatience mediated the association between psychiatric distress and POG in males, whereas the indirect effect of impatience was not significant in females.

**Conclusions:**

The results of this work highlight gender-related difference among online gamers in the mediation effect of impulsivity between psychiatric distress and POG and provide novel insights regarding clinical implications for preventing or treating POG.

## Introduction

### Background

Problematic online gaming (POG) may be defined as the persistent and recurrent use of the internet to play video games, leading to clinically significant impairment or distress. Internet gaming disorder was included as a “condition for further study” in the Diagnostic and Statistical Manual of Mental Disorders, Fifth Edition, section 3 [1]. Furthermore, gaming disorder, including online gaming, has been formally included along with gambling disorder as a disorder because of addictive behaviors in the recently released listing of conditions in the International Classification of Diseases 11th Revision [2]. Some have considered POG to be a global public health problem because of its relevant high prevalence and significant negative outcomes in worldwide populations, particularly among adolescents [3,4]. A recent review suggested that the overall prevalence of POG ranged from 0.7% to 15.6% in studies of naturalistic populations, with an average percentage of 4.7% [5].

Psychiatric symptoms such as depression have been associated more broadly with POG and problematic internet use [6,7]. POG has been positively correlated with psychiatric symptom dimensions including depressed mood, loneliness, social anxiety, and negative self-esteem [8-11]. Individuals (particularly females) may engage in potentially addictive behaviors to escape from negative mood states or other psychiatric distress (negative reinforcement motivations), and this may lead to problematic or addictive engagement. Although previous research has focused primarily on the direct association between psychiatric distress and POG, fewer studies have examined how specific factors may moderate relationships between psychiatric distress and POG or how the relationships between psychiatric distress and POG may operate through intervening variables via mediation effects. To address these gaps, this study constructed a moderated mediation model to test the mediating role of impulsivity and the moderating role of gender in the relation between psychiatric distress and POG. Such studies could advance our understanding of how and when psychiatric distress might lead to greater POG and may have implications for the prevention and treatment of POG.

### Impulsivity as a Mediator or Moderator

Impulsivity has been described as being characterized by “actions that are poorly conceived, prematurely expressed, unduly risky, or inappropriate to the situation and that often result in undesirable outcomes” [12]. Although impulsivity is a multidimensional construct, impatience is an important component of impulsiveness that may result from relative disregard of future outcomes and oversensitivity to immediate rewards [13]. Impulsivity has been related to substance use, gambling, and gaming [14]. Impulsivity has been proposed to contribute significantly to the development and/or maintenance of addictions [3,15]. It has also been linked to poor addiction treatment outcomes [16]. Furthermore, impulsivity has been associated with problematic internet use and POG. For instance, adolescents with internet addiction exhibited more impulsivity than those without [17], and the severity of internet addiction was positively correlated with the level of impulsivity in individuals with internet addiction [18]. Greater impulsivity among internet users was associated with poorer control over the use of the internet [19]. Similarly, individuals with POG have been found to score high on measures of impulsivity [20].

Psychiatric symptoms may relate to impulsivity. Negative emotional states may lead individuals to focus more on their feelings, which may trigger poor self-control [21]. Semple et al [22] found that depression scores could best discriminate between substance users with high and low impulsivity. Depression and loneliness scores have also correlated with low self-control and problematic internet use [6].

Previous studies have shown that impulsivity may mediate the relation between psychiatric distress and addictive behaviors. For example, impulsivity has been shown to mediate the influence of life stress on pathological gambling [23] and the relationship between depression and problematic gambling [24]. However, mixed findings have been reported in moderation analyses. Although a moderating effect of impulsivity was also observed in a study by Tang and Wu [23], in which a positive association between life stress and pathological gambling was significant among those with low impulsivity only, and pathological gambling remained high regardless of the stress level among those with high impulsivity, this moderating effect was not supported in the study by Clarke [24]. Although studies have examined the relationship between impulsivity and problematic internet use [17,18], and the mediating and/or moderating effects of impulsivity on psychiatric distress and pathological gambling [23,24], few studies have examined a role for impulsivity in relationships between psychiatric distress and POG. Internet addiction has been reported to have comparable levels of impulsivity compared with pathological gambling [18], and POG may be characterized by high impulsivity such as other addictive disorders including gambling disorder [20]. Therefore, previous studies of pathological gambling may shed light on mechanisms of how psychiatric distress may relate to POG. Thus, this study investigated the extent to which impulsivity would mediate and/or moderate a hypothesized relationship between psychiatric distress and POG. On the basis of the findings above, we proposed the following hypotheses (denoted H#):

- **H1:** POG would correlate positively with impulsivity with a moderate effect size (H1a) and psychiatric distress with a large effect size (H1b).
- **H2:** According to the mediation model, impulsivity would mediate the relationship between psychiatric distress and POG. Psychiatric distress would relate to POG directly as well as indirectly through impulsivity.
- **H3:** According to the moderation model, impulsivity would moderate the relation between psychiatric distress and POG, with the relationship being stronger for individuals with lower impulsivity.

### Gender as a Moderator

Although psychiatric distress may relate to impulsivity and POG, it is possible that not all individuals are equally inﬂuenced. This study examined whether the direct effect of psychiatric distress on POG and the indirect effect of impulsivity would be moderated by gender.

**Figure 1:**
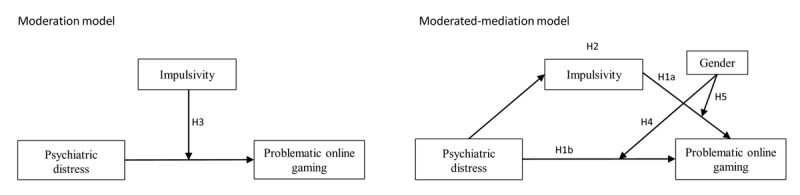
Conceptual model of hypotheses. H1a: Hypothesis 1a; H1b: Hypothesis 1b; H2: Hypothesis 2; H3: Hypothesis 3; H4: Hypothesis 4; H5: Hypothesis 5.

Gender may relate importantly to internet gaming behaviors. First, boys have been found to be more likely than girls to report having played video games [25-27] and to report more problematic gaming than girls [28]. Second, being male was a strong predictor of problematic game use [26], and POG was strongly associated with being male [29]. Third, gender-related differences may extend to gaming motivation. Female gamers scored significantly higher on fantasy, escape, social, and recreation motives, whereas male gamers reported significantly higher scores on competition motives [9]. Male players also reported a greater need for gaming-related friendships than did females [30].

Gender-related differences may also exist in impulsivity’s relationship to addictive behaviors. Gender was found to moderate the relationship between sensation seeking/impulsivity and alcohol use; in particular, males and females were found to have comparable alcohol use frequencies under conditions of low sensation seeking/impulsivity, and males were found to have a higher frequency of alcohol use than females under conditions of high sensation seeking/impulsivity [31]. A moderating effect of gender on the association between impulsivity and alcohol-use problems was also found in the study by Stoltenberg et al [32]. Similarly, impulsivity was found only to associate with levels of alcohol use in males [33]. Given that urges/cravings in POG and substance-use disorders may share similar neurobiological mechanisms [34], gender-related differences in previous studies of alcohol use may extend to POG, and this possibility should be tested directly.

Together, both the association between psychiatric distress and POG and the association between impulsivity and POG may be moderated by gender. Furthermore, if impulsivity were to mediate the relation between psychiatric distress and POG, and gender were to moderate the association between impulsivity and POG simultaneously, the mediating effect of impulsivity would be moderated by gender. In other words, there would be a moderated-mediation model involving impulsivity and gender in the relation between psychiatric distress and POG. We sought to test this model. Thus, we put forward the following hypotheses (see Figure 1).

- **H4:** Gender would moderate the relationship between psychiatric distress and POG, with the relation being stronger for males.
- **H5:** Gender would moderate the mediating effect of impulsivity in the relationship between psychiatric distress and POG, with the mediating effect only being significant among males*.*

## Methods

### Participants and Procedure

We used data from a nationwide Hungarian online gaming sample. Participants were recruited via gaming-related websites and Facebook pages. A total of 3 calls containing the link to the questionnaire were posted on a popular gaming magazine’s (ie, GameStar) website and Facebook page in August and September 2014. The magazine’s Facebook page had approximately 65,000 followers at the time of the data collection. The post was liked more than 800 times and shared more than 130 times during this period, reaching a wide circle of Hungarian gamers. A shopping voucher of approximately 300 Euro was drawn and offered to 1 study participant as an incentive to boost participation. For more details of the data collection process, see the study by Király O et al [35]. Participants were informed about the aim of the study, the time necessary for completion, and confidentiality of the data. Every participant included in this analysis provided informed consent by ticking a checkbox indicating agreement before starting the questionnaire. Participants aged between 14 and 17 years needed to indicate the approval of their parents with an affirmative response from the parent to the question stating, “I allow my child to participate: parent.” This study was approved by the Institutional Review Board of Eötvös Loránd University, Budapest, Hungary. The Institutional Review Board of Yale University approved the use of deidentified data in these analyses. This study was performed in line with the Helsinki Declaration. We used the larger sample in 2 previous studies, which did not include data analysis findings reported in this study [35,36].

A total of 7757 gamers started the survey. To verify unique entries (ie, emanating from different individuals), email addresses were checked for uniqueness, and cases having the same email address were removed from the dataset. Furthermore, we checked for invalid answer patterns (eg, the impulsivity scale had several reversed items—if a person gave the same values for all items, the answers were considered as invalid). We deleted responses from individuals with invalid answer patterns. After excluding cases reflecting duplicate submissions, severe incompleteness or inconsistencies on the variables from this study, 5222 online gamers remained in the sample from this study (4830 males and 384 females). Due to the overrepresentation of males that is frequent in specific video gamer samples, we identified a matched sample of males based on the female participants (298 males and 298 females) for further analysis. The matching method is described in the Statistical Analysis section. In the sample from this study, the mean age was 21.4 years (SD 4.5, age range 14-38). Most participants were either single (41.6%, 248/596) or in a relationship but living separately (39.3%, 234/596), and most were students (68.9%, 409/594). For further details on sample demographics, see Table 1.

### Measures

#### Problematic Online Gaming Questionnaire

The Problematic Online Gaming Questionnaire (POGQ) is an 18-item scale assessing POG [37], showing good psychometric properties in both adult and adolescent samples (Pápay et al). The scale comprises 6 factors: social isolation, interpersonal conflicts, overuse, withdrawal, immersion, and preoccupation. Participants responded on a 5-point Likert scale (1=“never” and 5=“almost always/always”) to each item (eg, “How often do you get irritable or upset when you cannot play?” and “How often do you neglect other activities because you would rather be gaming?”), with higher scores indicating greater POG. The scale was originally developed in the Hungarian context and showed good psychometric properties in other cultures as well, for example, the study by Ballabio et al [10]. The internal consistency of this scale was 0.89 in this study.

#### Brief Symptom Inventory

The Brief Symptom Inventory assesses psychiatric distress with 53 items on 9 self-reported clinically relevant psychological symptoms: somatization, obsession-compulsion, interpersonal sensitivity, depression, anxiety, hostility, phobic anxiety, paranoid ideation, and psychoticism [38]. Respondents rank the distress level of each item (eg, “Having to check and double check what you do,” “Feeling fearful,” and “Trouble falling asleep”) on a 5-point Likert scale (0=“not at all” and 4=“extremely”) during the past 7 days. Rankings characterize the intensity of distress during the past 7 days. In this study, a global index of *Global Severity Index (GSI)*, namely the mean for all 53 items, was used to assess the level of general distress. Higher GSI scores indicate stronger psychiatric distress. The scale was previously adapted to the local (Hungarian) culture and showed good psychometric properties [39]. The internal consistency of this scale was 0.96.

**Table 1:** Demographics and weekly gaming time of sample participants.

Demographics		Male (n=298)	Female (n=298)
Age in years, mean (SD)		21.4 (4.5)	21.4 (4.5)
Education (years completed), mean (SD)		13.0 (2.6)	13.0 (2.6)
**Marital status, n (%)**			
	Single	124 (41.6)	124 (41.6)
	In a relationship but living separately	117 (39.3)	117 (39.3)
	Living in a partnership	51 (17.1)	51 (17.1)
	Married	6 (2.0)	6 (2.0)
	Divorced	0 (0.0)	0 (0.0)
	Widowed	0 (0.0)	0 (0.0)
Currently a student^a^, n (%)		203 (68.6)	206 (69.1)
**Working status, n (%)**			
	Does not work	165 (55.4)	165 (55.4)
	Has a part-time job	58 (19.5)	58 (19.5)
	Has a full-time job	75 (25.2)	75 (25.2)
**Weekly gaming time^b^, n (%)**			
	Less than 7 hours	48 (16.2)	57 (19.2)
	7-14 hours	52 (17.5)	78 (26.3)
	15-28 hours	109 (36.7)	100 (33.7)
	29-42 hours	57 (19.2)	49 (16.5)
	More than 42 hours	31 (10.4)	13 (4.4)

^a^2 missing values in males.

^b^2 missing values (1 male and 1 female, respectively).

#### Barratt Impulsiveness Scale

The Barratt Impulsiveness Scale (BIS)-21, revised from the original BIS [40], has been tested in 3 different Hungarian adult samples, including a representative one, and assesses impulsivity with 21 items comprising 3 components of impulsivity: self-control, impulsive behavior, and impatience [41]. Participants indicated their responses on a 4-point Likert scale (1=“rarely/never” and 4=“almost always/always”) to each item (eg, “I am future oriented,” “I do things without thinking,” and “I am restless at the theater or lectures,”), with higher scores indicating greater impulsivity. The internal consistency (Cronbach alpha) was .74 for the self-control subscale, .78 for the impulsive behavior subscale, and .63 for the impatience subscale, whereas it was .80 for the whole scale.

### Statistical Analysis

We used the case-control matching tools in Medcalc v17.8 (MedCalc Software bvba) to obtain matched data from the sample pool (N=5222). Female status was set to be the matching target group, and male cases were chosen from the pool to match each female case by case. The matching condition was set to include a maximal allowable difference in age of 1 year and required both paired individuals to be the same on measures of education, marital relationship, and work status. This approach generated 298 pairs of cases satisfying the matching conditions.

Description analysis, correlation analysis, and *t* tests were performed with SPSS 19. Internal consistencies were assessed by Cronbach alpha coefficient. Cohen *d* was used to measure the effect size.

Mediation and moderation effects were tested using SPSS PROCESS (v3.0) for bootstrapping as described by Hayes [42]. PROCESS is a computational tool for path analysis–based moderation and mediation analysis as well as for their combination [42]. We first tested a mediation model (model number 4 in PROCESS) for H2 using the total score of the BIS-21; we then tested a moderation model (model number 1 in PROCESS) for H3. If positive mediation and/or moderation effects of impulsivity were observed, then a series of post hoc analyses were used to examine specific subscales of the BIS-21 separately. We further tested the moderated mediation model (model number 15 in PROCESS) for H4 and H5. Values of variables were standardized before calculating the models in PROCESS for the purpose of obtaining the standardized regression coefficient. Age, education, and working status were included as covariates in all models. Signiﬁcant interactions in the moderated-mediation model were followed-up with simple slopes analysis at high (+1 SD) and low (−1 SD) values of the moderator variable [42]. Indirect mediating effects were evaluated with 95% CIs using the percentile method based on 5000 bootstrap samples. If the CI did not contain zero, then the indirect effect was considered statistically signiﬁcant [42]. If the presence of such an indirect effect depended on the value of a moderating variable, then it was considered an indication of moderated mediation.

## Results

### Sample Description

Given the matching process, males and females in the subsample had precisely the same demographic characteristics with respect to age, education, marital status, and working status. Student status was also largely similar. Chi-square testing revealed that a higher percentage of males played games for more than 14 hours per week compared with females (χ^2^_4_=14.3; *P*=.006, odds ratio=1.64).

The descriptive statistics and zero-order correlations for demographic variables and psychological measures are presented in Table 2. GSI was positively and strongly correlated with POG (*r*=0.52, *P*<.001), whereas impulsivity was moderately related to POG (*r*=0.33, *P*<.001); thus, H1 was confirmed.

### Gender-Related Differences

We examined gender-related differences using *t* tests (see Table 3). Statistically significant gender-related differences were not observed in impulsivity. Females scored higher in immersion (*t*_565_=−3.52; *P<*.001, Cohen *d*=0.30) but lower in overuse (*t*_563_=2.10; *P=*.04, Cohen *d*=−0.18) than males on subscales of the POGQ. GSI scores were higher in females than males (*t*_491_=−5.40; *P<*.001, Cohen *d*=0.49), but males spent more weekly time gaming than did females (*t*_592_=3.14; *P=*.002, Cohen *d*=−0.26).

**Table 2:** Mean, SD, and Spearman’s correlation matrix with *P* values of overall variables.

Variables	1	2	3	4	5	6	7	8	9
1. Age	1.00	—^a^	—	—	—	—	—	—	—
2. Education	0.66 (<.001)	1.00	—	—	—	—	—	—	—
3. Working status^b^	.64 (<.001)	.42 (<.001)	1.00	—	—	—	—	—	—
4. Problematic online gaming	−0.18 (<.001)	−0.16 (<.001)	−0.18 (<.001)	1.00	—	—	—	—	—
5. Global Severity Index	−0.20 (<.001)	−0.21 (<.001)	−0.15 (<.001)	0.52 (<.001)	1.00	—	—	—	—
6. BIS^c^ self-control	−0.06 (=.17)	−0.06 (=.18)	−0.08 (=.07)	0.19 (<.001)	0.16 (<.001)	1.00	—	—	—
7. BIS impulsive behavior	−0.09 (=.03)	−0.12 (=.006)	−0.04 (=.38)	0.25 (<.001)	0.13 (<.001)	0.37 (<.001)	1.00	—	—
8. BIS impatience	−0.06 (=.14)	−0.03 (=.43)	−0.03 (=.44)	0.36 (<.001)	0.33 (<.001)	0.22 (<.001)	0.43 (<.001)	1.00	—
9. BIS total score	−0.09 (=.04)	−0.09 (=.047)	−0.06 (=.15)	0.33 (<.001)	0.27 (<.001)	0.78 (<.001)	0.76 (<.001)	0.70 (<.001)	1.00
Mean (SD)	21.38 (4.48)	13.00 (2.55)	1.70 (0.85)	0.74 (0.66)	12.8 (3.76)	17.37 (4.40)	9.96 (3.06)	12.56 (3.43)	39.70 (8.02)

^a^The correlation coefficient was not shown as it was shown in the asymmetrically diagonal position of the table.

^b^Working status: it was coded as 1=“does not work,” 2=“has a part-time job,” and 3=“has a full job.”

^c^BIS: Barratt Impulsiveness Scale (version 21).

**Table 3:** Gender differences in overall variables.

Variables		Male, mean (SD)	Female, mean (SD)	*t* test(df)	*P* value	Cohen *d*
**Impulsivity**						
	Self-control	17.15 (4.34)	17.60 (4.46)	−1.18(*539*)	.24	0.10
	Impulsive behavior	9.82 (3.11)	10.09 (3.00)	−1.03(*547*)	.30	0.09
	Impatience	12.42 (3.43)	12.69 (2.43)	−0.89(*545*)	.37	0.08
	Total score	39.22 (7.99)	40.18 (8.04)	−1.38(*526*)	.17	0.12
**Problematic online gaming**						
	Preoccupation	5.24 (1.83)	5.33 (2.03)	−0.56(*572*)	.57	0.05
	Immersion	11.87 (3.51)	12.93 (3.65)	−3.52(*565*)	<.001	0.30
	Withdrawal	6.89 (3.24)	6.85 (3.44)	0.16(*566*)	.87	−0.01
	Overuse	6.05 (2.67)	5.58 (2.72)	2.10(*563*)	.04	−0.18
	Social isolation	5.08 (2.40)	5.09 (2.70)	−0.03(*570*)	.98	0.00
	Total score	39.22 (11.15)	39.73 (11.74)	−0.53(*540*)	.60	0.04
	Global Severity Index	0.58 (0.55)	0.89 (0.73)	−5.40(*491*)	<.001	0.49
	Weekly game time^a^	2.90 (1.19)	2.61 (1.10)	3.14(*592*)	.002	−0.26

^a^6-point scale: 0=“none,” 1=“less than 7 hours,” 2=“7-14 hours,” 3=“15-28 hours,” 4=“29-42 hours,” and 5=“more than 42 hours.”

**Figure 2:**
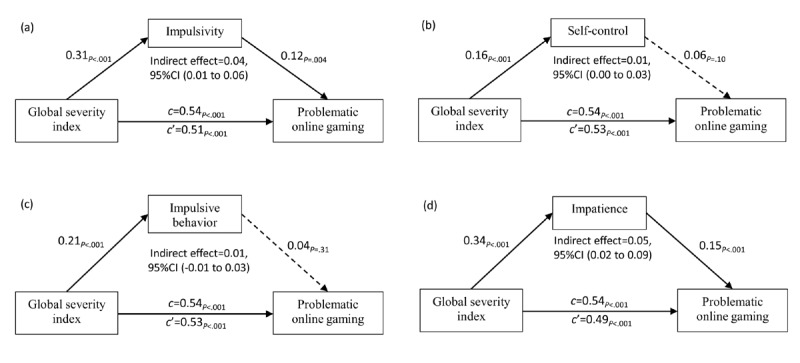
Results of models testing for mediating effects of impulsivity and its dimensions in the relationship between measures of global severity index and problematic online gaming (N=5000 bootstrapping resamples). Path c=total (nonmediated) effect; Path c'=direct (controlling mediator) effect; all paths are quantified with standardized regression coefficients.

**Table 4:** Summary of multiple regression analyses for the moderated analysis.

Predictors^a^	Beta	*SE*	*t* test(*df*)	*P* value	95% bootstrap CI
Age^b^	−.06	0.06	−1.04(*492*)	.30	−0.18 to 0.06
Education^b^	−.06	0.05	−1.25(*492*)	.21	−0.16 to 0.04
Work status^b^	.03	0.05	0.60(*492*)	.55	−0.07 to 0.13
GSI^c^	.53	0.04	12.52(*492*)	<.001	0.44 to 0.61
Impulsivity	.12	0.04	3.02(*492*)	.003	0.04 to 0.20
GSI×Impulsivity	−.06	0.04	−1.64(*492*)	.10	−0.13 to 0.01

^a^Dependent variable is POG (problematic online gaming).

^b^Covariate variables; N=5000 bootstrapping resamples.

^c^GSI: Global Severity Index.

### Impulsivity

To test the hypothesis regarding a mediating role for impulsivity (H2), the mediating effect of impulsivity as well as indirect effects and direct effects of psychiatric distress on POG was calculated with 5000 bootstrap samples. Age, education, and working status were controlled as covariates. The bootstrap results showed that impulsivity partially mediated the effect of GSI on POG. In particular, the total (nonmediated) effect of GSI was significant and strong (beta=.54, SE 0.04, *t*_*465*_=13.95, *P<*.001). After controlling for impulsivity, the direct effect of psychiatric distress was weakened but remained significant and strong (beta=.51, SE 0.04, *t*_*464*_=12.50, *P<*.001). The indirect effect of GSI through impulsivity was significant, with a small effect size estimate (0.04 with a 95% bootstrap CI of 0.01 to 0.06; see model a in Figure 2).

In post hoc analyses, we examined the specific subscales of the BIS-21 in mediation analyses. In the model of self-control and impulsive behavior (see model b and c in Figure 2), the 95% bootstrap CI of the indirect effect included zero, so the mediation effect was not significant. However, the mediation effect of impatience was significant with an effect size estimate of 0.05 with a 95% bootstrap CI of 0.02 to 0.09 (see model d in Figure 2).

To test the moderation effect of impulsivity (H3), a multiple regression analysis was conducted to determine main and interaction effects of impulsivity and GSI on POG. As shown in Table 4, main effects of GSI (beta=.53, *P*<.001) and impulsivity (beta=.12, *P*=.003) were both significant, but their interaction term (GSI×Impulsivity) was not significant (*P*=.10). Thus, the moderation effect of impulsivity was not supported in the study.

### Test of Moderated Mediation

As only the impatience subscale of BIS-21 was significant in the mediation model, we further tested H4 and H5 with respect to impatience by adding gender as a moderator into the model. The result revealed that gender significantly moderated the relationship between impatience and POG (beta=.19, *P*=.02), but its moderation effect in the direct path between GSI and POG was not significant (beta=.08, *P*=.34; see Table 5).

**Table 5:** Summary of multiple regression analyses for the moderated mediation analysis.

Dependent variables		Beta	*SE*	*t* test(*df*)	*P* value	95% bootstrap CI
**Impatience**						
	Age^a^	−.02	0.07	−0.23(*464*)	.82	−0.15 to 0.12
	Education^a^	.02	0.06	0.27(*464*)	.79	−0.10 to 0.13
	Work status^a^	.04	0.06	0.69(*464*)	.49	−0.07 to 0.15
	GSI^b^	.34	0.04	7.52(*464*)	<.001	0.25 to 0.42
**Problematic online gaming**						
	Age^a^	−.04	0.06	−0.73(*460*)	.47	−0.16 to 0.07
	Education^a^	−.07	0.05	−1.50(*460*)	.14	−0.17 to 0.02
	Work status^a^	.02	0.05	0.32(*460*)	.75	−0.08 to 0.11
	GSI	.53	0.04	12.32(*460*)	<.001	0.44 to 0.61
	Impatience	.14	0.04	3.64(*460*)	<.001	0.07 to 0.22
	Gender	.17	0.08	2.25(*460*)	.03	0.02 to 0.32
	Gender×GSI	.08	0.08	0.96(*460*)	.34	−0.08 to 0.25
	Gender×Impatience	.19	0.08	2.35(*460*)	.02	0.03 to 0.34

^a^Covariate variables; N=5000 bootstrapping resamples.

^b^GSI: Global Severity Index.

**Figure 3:**
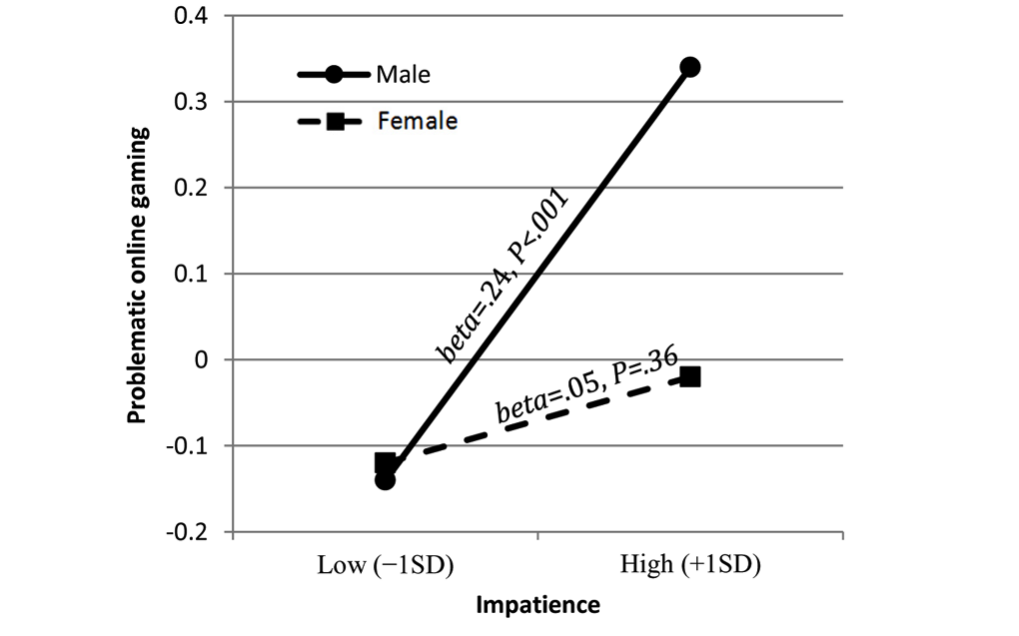
Simple slopes probing the interaction between gender and impatience in the prediction of problematic online gaming (Z score).

Graphs of the interaction (Gender×Impatience) are presented in Figure 3 with simple slopes, derived from the regression equations, where high and low values of impatience were speciﬁed as 1 SD above and below the mean. Results indicate a signiﬁcant positive association between impatience and POG for males (beta=.24, *P*<.001) but not for females (beta=.05, *P*=.36). In females, impatience and POG were not associated. Testing the indirect effect of psychiatric distress on POG via impulsivity revealed that impatience scores mediated the association between GSI and POG but only in males and not in females (see Table 6).

**Table 6:** Conditional direct and indirect effects of Global Severity Index (independent variable) on Problematic Online Gaming (outcome variable) via impatience (mediator) and gender (moderator).

Values of the moderator	Direct effects				Indirect effects		
	Beta	SE	*t* test(*df*)	*P* value	Effect (bootstrap estimate)	SE (bootstrap estimate)	95% bootstrap CI
Male	.57	0.07	8.41(*496*)	<.001	0.08	0.02	0.04 to 0.13
Female	.49	0.05	9.42(*496*)	<.001	0.02	0.02	−0.02 to 0.06

## Discussion

### Principal Findings

Although several studies have examined links between psychiatric distress and POG [8-11], this study is the ﬁrst to provide empirical evidence investigating the extent to which impulsivity and facets thereof may mediate and/or moderate this relationship in a gender-sensitive fashion. Our a priori hypotheses were partially supported in which impulsivity mediated the relationship between psychiatric distress and POG. However, this relationship seemed most relevant for impatience and was moderated by gender such that the mediating relationship was evident in males but not females. These findings provide insight into possible mechanisms by which psychiatric distress may influence POG.

The ﬁnding that POG was related to psychiatric distress supported H1 and is consistent with previous findings in POG [8-10,43], as well as ﬁndings in other behavioral addictions [44,45]. The findings from this study expand upon previous ones through exploration of a role for impulsivity. As hypothesized (H2), the relationship between psychological distress and POG was mediated partially through impulsivity, consistent with studies of problematic gambling [23,24]. However, the hypothesized moderation effect of impulsivity was not observed, consistent with findings from the study by Clarke [24] but not from the study by Tang et al [23]. For POG, psychological distress and impulsivity did not interact to account for POG beyond the main effects. As distress increased, the likelihood of experiencing POG symptoms increased, partially through impulsivity. The results resonate with the pathways model of problematic gambling described by Blaszczynski et al [46]. According to the third path in the model, the effect of impulsivity may be increased when experiencing negative emotions or when feeling pressured or stressed, and impulsivity is proposed to mediate the effects of emotional disturbances on problem and pathological gambling symptoms through an interactive process [46]. Further analysis revealed that this mediation effect only appeared in the dimension of impatience. Previous studies have found that impatience is related to increased substance use [47], pathological gambling [48], and POG [49]. The findings from this study suggest an important role of psychiatric distress that may influence POG indirectly through impatience; therefore, helping to develop skills in specific impatience-related domains (emotion regulation and behavioral control) may be important when treating patients with POG.

Earlier studies have reported that boys were more likely to play online games than girls and also more likely to be problematic players [11,28]. Our results indicate that males did spend more time online playing games compared with females; however, there was no gender-related difference in overall POG (as assessed by POGQ total scores) in the sample from this study. Possible reasons may be related to the sample from this study, which was recruited from gaming-related websites and Facebook pages; therefore, both gender groups may have comprised highly engaged gamers [50]. In this study, females had higher psychological distress than males, but the groups did not differ in levels of impulsivity. Moreover, results provided some support for gender as a moderator among the association between impatience and POG in the mediation model. Specifically, as predicted in H5, the indirect effect of impatience was significant in males but not in females. The result is consistent with previous findings in alcohol-use behaviors, in which impulsivity was correlated with alcohol use in males but not in females [33]. The ﬁndings from this study suggest that males may be more vulnerable to POG triggered by impulsivity. However, gender did not moderate the direct effect of psychological distress and POG as hypothesized in H4, which suggests both males and females may exhibit POG symptoms in relation to psychiatric distress.

### Limitations and Implications

This research should be viewed in light of several limitations. First, male players are overrepresented (92.5%) in the original sample of gamers (N=5222) recruited online. Therefore, a new matched sample based on the female cohort was used for this study, and the results may not extend to the general population. Second, this study involved cross-sectional survey data. Thus, it does not permit identification of cause-and-effect associations. For example, we could not determine whether psychiatric distress increased POG or whether POG led to distress. It is also possible that there was a reciprocal inﬂuence between distress and POG. Future studies should utilize longitudinal methods to examine the directionality of relationships among POG, psychiatric distress, and impulsivity. Third, despite the advantage of a larger sample size, the self-selected data collected online from a Hungarian sample are derived from a convenience sample, thus limiting the generalizability of our findings. The sample likely overrepresents active and highly engaged gamers, as suggested elsewhere [50]. Nevertheless, data from this sample have both limitations and strengths and may be particularly suitable for examining potential roles of impulsivity and psychiatric distress in problematic gaming. The use of different anchor points for Likert scales across instruments in the study also has both strengths and limitations, with strengths including the use of values employed in the originally described and validated instruments and weaknesses including potential complexities in comparing findings internally across instruments. Finally, the self-report nature of the data may introduce certain biases (eg, memory recall bias and social desirability bias) that should be considered.

Despite the above limitations, the results have significant clinical implications. Cooccurring features of POG (eg, depression and impulsivity) should be considered in its treatment [51]. The findings in this investigation suggest that because distress affects impulsivity, it may thus be appropriate to treat emotional distress experienced by problematic gamers in addition to treating impulsivity, as suggested by Clarke [24]. Moreover, targeting impulsivity, and particularly impatience, may be helpful to weaken the link between psychiatric distress and POG, especially for males. Cognitive-behavioral therapy, by itself or in conjunction with medication, might be helpful in treating associated impatience/restlessness and emotional distress [52]. As video games are attractive to and accepted by those at risk of POG, they may be considered in novel approaches to help control impulsivity and improve emotion regulation. For example, in other disorders, some video games (eg, PlayMancer) may be effective as additional therapy tools to help improve emotional regulation and impulse control in cases with bulimia nervosa [53] and gambling disorder [54]. The potential therapeutic effect of video games in POG may be worth exploring in future studies. However, one should also consider the potential triggering effects of exposing individuals with POG to online games, especially if abstinence is being targeted. As males with high impulsivity may be more vulnerable to POG than females, future interventions for POG should consider gender-related differences in this domain. Furthermore, given that attention-deficit/hyperactivity disorder (ADHD) has been linked to online gaming [55] and greater impatience/restlessness during abstinence from gaming [56], medications that reduce impulsive behaviors (eg, stimulants like methylphenidate or nonstimulants like atomoxetine) may be helpful in reducing POG, particularly in males. As relationships between ADHD and problematic internet use, more broadly, have been observed, especially in young adults [57], the extent to which the findings and corresponding intervention-related considerations are relevant to a broader range of problematic online behaviors (gambling, shopping, and pornography viewing) warrants direct investigation. Among females, the higher GSI scores suggest that psychopathology may be a greater consideration related to POG in females as compared with males. Given that females exhibit anxiety more frequently than males, they receive more mental health treatment and engage in addictive behaviors like gambling for negative reinforcement motivations [58-60]. Future studies should assess these domains as they relate to POG, particularly in females.

### Conclusions

In conclusion, this study provides some of the first empirical data investigating the extent to which impulsivity and its dimensions may mediate and/or moderate relationships between psychiatric distress and POG in a gender-sensitive fashion. The study suggests that impulsivity (specifically impatience) acts as a mediator rather than a moderator in the relationship between psychiatric distress and POG. A moderating effect of gender was not found in the direct relationship between psychiatric distress and POG. However, a moderated mediation analysis suggested that impatience mediated the association between psychiatric distress and POG in males, whereas the indirect effect of impatience was not significant in females. The findings suggest important implications for preventing or treating POG in online gamers. Future studies should examine other individual differences (eg, with respect to age or race/ethnicity) that may also help understand different populations and potentially advance prevention and treatment efforts.

## Abbreviations

ADHD: attention-deficit/hyperactivity disorder
BIS: Barratt Impulsiveness Scale
GSI: Global Severity Index
POG: problematic online gaming
POGQ: Problematic Online Gaming Questionnaire
